# A telomere-related signature for predicting prognosis and assessing immune microenvironment in osteosarcoma

**DOI:** 10.3389/fphar.2024.1532610

**Published:** 2025-01-27

**Authors:** Shihao Li, Lina Zhang, Haiyang Zhang

**Affiliations:** ^1^ Department of Orthopedics, Zibo Central Hospital West Campus, Zibo, China; ^2^ Department of Hand and Foot Surgery, Zibo Central Hospital, Zibo, China

**Keywords:** osteosarcoma, telomere-related signature, prognosis, immune microenvironment, drug sensitivity, MAP7

## Abstract

**Objective:**

Osteosarcoma is the most common primary bone cancer with a high propensity for local invasion and metastasis. An increasing number of research studies show that telomeres play an important role in the occurrence and development of cancer. Thus, we established a telomere-related signature in osteosarcoma to comprehensively evaluate the pathogenic roles of telomeres in this disease.

**Methods:**

The data on osteosarcoma were collected from the TARGET and Gene Expression Omnibus databases. First, we constructed a telomere-related signature using univariate and LASSO Cox regression analyses. Subsequently, we analyzed the prognostic value, functional annotation, immune microenvironment, and cell communication patterns of the telomere-related signature in osteosarcoma via comprehensive bioinformatics analyses. Cell proliferation was analyzed using the CCK-8 assay, and cell migration and invasion capabilities were evaluated using the Transwell assay.

**Results:**

Based on the SP110, HHAT, TUBB, MORC4, TERT, PPARG, MAP3K5, PAGE5, MAP7, and CAMK1G, a telomere-related signature was built in osteosarcoma patients. The telomere-related signature could effectively predict the prognosis of osteosarcoma patients. The osteosarcoma patients in the high TELscore group exhibited poor prognosis. In addition, the telomere-related signature demonstrated predictive value for the immune microenvironment and drug sensitivity in osteosarcoma. Finally, we discovered significant reduction in MAP7 expression in osteosarcoma cells, and patients with low MAP7 expression had poor prognosis. Moreover, the overexpression of MAP7 significantly reduced cell proliferation, the ability of cell migration, and invasion in osteosarcoma cells.

**Conclusion:**

A telomere-related signature was constructed in osteosarcoma patients, offering predictive values into prognosis, the immune microenvironment, and drug sensitivity. Moreover, MAP7 might serve as a prognostic marker for osteosarcoma patients.

## 1 Introduction

Osteosarcoma is a primary malignant bone tumor that arises from interosseous leaf cells. Osteosarcoma most often occurs near the metaphyseal plates of the long bones. The most common sites are the femur (42%), tibia (19%), and humerus (10%). Other prevalent sites of occurrence include the craniofacial bones (8%) and the pelvis (8%), with primary osteosarcoma of extragnathic craniofacial bones accounting for 2% of all osteosarcomas (Cho et al., 2014). It is characterized by the rapid proliferation of tumor cells, which directly leads to the formation of immature bone or bone-like tissue (Xu et al., 2019). As the most prevalent kind of primary malignant bone tumor in children and adolescents (Kebudi et al., 2016; Pastorino et al., 2023), osteosarcoma accounts for 2.4% of all juvenile cancers and approximately 20% of all primary bone tumors (Ottaviani and Jaffe, 2009). Osteosarcoma is very prone to metastasis in its early stages, with the lungs being the most common metastatic location, followed by the distal bones and lymph nodes (Cersosimo et al., 2020). In recent years, reactive oxygen species (ROS)-mediated therapy, such as photodynamic therapy (PDT), has emerged as a new promising treatment method for osteosarcoma (Tan et al., 2022). Nanoparticles (CI@HSA NPs) encapsulating capsaicin (CAP) and the photosensitizer IR780 promote ferroptosis in osteosarcoma and improve the hypoxic microenvironment by releasing capsaicin, thereby improving the efficacy of photodynamic therapy (PDT) in osteosarcoma (Wang et al., 2024). However, in the early stage of osteosarcoma, there is a lack of diagnostic markers and therapeutic targets. Thus, the osteosarcoma patients have a poor prognosis (Sugito and Kamal, 2022).

Telomeres are specialized structures that form at the ends of chromosomes, serving to protect them from nucleolytic degradation and distinguish them from DNA double-strand breaks (Fajkus et al., 2005). Previous studies have indicated that telomere anomalies may lead to a variety of disorders, including cancer, cardiovascular disease, myelodysplastic syndrome, dyskeratosis congenita, and Revesz syndrome (Maciejowski and de Lange, 2017; Savage, 2018; Armanios, 2022). Ennour-Idrissi et al. (2017) demonstrated that longer telomeres are associated with better outcomes for breast cancer patients. Mirabello et al. (2011) found that female individuals with short telomeres may be predisposed to osteosarcoma. In telomerase-negative osteosarcoma U2OS cells, the deletion of the telomere-binding protein TPP1 can lead to telomere shortening, increased apoptosis, and heightened sensitivity to radiation (Qiang et al., 2014). Furthermore, in both osteosarcoma Saos-2 and U2OS cell lines, the knockdown of pinX1, an intrinsic telomerase inhibitor and a putative tumor suppressor gene, results in telomere shortening, increased apoptosis, and enhanced sensitivity to ionizing radiation (Li et al., 2015). Notably, oxidative stress caused by excess ROS accelerates telomere shortening (Fouquerel et al., 2019). The photosensitizer TMPipEOPP selectively binds to telomeric DNA G-quadruplex and cleaves it upon photo-irradiation through ROS production, leading to cancer cell death (Zhu et al., 2016). Therefore, further exploration of telomere-related genes in osteosarcoma is essential for early diagnosis and improved treatment strategies.

Previous research has primarily focused on telomere length in cancer and its role in the prognosis of osteosarcoma. However, the impact of telomere-related genes on the progression of osteosarcoma has not been explored. Consequently, we developed a telomere-related signature based on these genes to predict the outcomes and investigate the potential implications of this signature in the immune microenvironment and drug sensitivity of osteosarcoma.

## 2 Methods

### 2.1 Data extraction

In the TARGET database (https://ocg.cancer.gov/programs/target), a total of 88 osteosarcoma samples with transcriptome data and clinical information were downloaded. After removing samples with incomplete clinical information, 84 samples with complete survival data (including overall survival and last survival status) were selected as the training set. The data were downloaded in matrix form, allowing for direct analysis without the need for preprocessing.

In the Gene Expression Omnibus database (GEO, https://www.ncbi.nlm.nih.gov/geo/), the GSE21257, GSE152048, GSE16088, GSE28424, GSE39055, and GSE16091 datasets were downloaded. The GSE21257 dataset consisted of 53 osteosarcoma samples and was downloaded in matrix form. The probes can be converted into gene symbols corresponding to the platform information and can be used directly for analysis. GSE16088 comprised 14 osteosarcoma samples and six normal samples, while GSE28424 included 19 osteosarcoma samples and four normal samples. GSE39055 contained 37 osteosarcoma samples along with complete clinical data, and GSE16091 included 34 osteosarcoma samples with comprehensive clinical data. A total of 2086 telomere-related genes were collected from the TelNet (http://www.cancertelsys.org/telnet/, Supplementary Table S1) (Li Q. et al., 2022). GSE152048 contained single-cell sequencing data of seven patients with osteosarcoma, from whom two samples, BC16 and BC21, were selected for subsequent analysis. The raw data of each sample of GSE152048 were processed using the software program Cell Ranger (https://www.10xgenomics.com/support/software/cell-ranger/, version6.0.2) to obtain the three files: barcodes.tsv, genes.tsv, and matrix.tsv. We downloaded these three files for each sample and used them directly for subsequent analysis without preprocessing.

### 2.2 LASSO Cox regression analysis

Univariate Cox regression analysis was performed on telomere-related genes, and genes significantly correlated with the prognosis of osteosarcoma patients were selected using a threshold of P < 0.01. Subsequently, LASSO Cox regression analysis was performed using the glmnet package (version 4.1.7) (Friedman et al., 2010) in R language to further optimize the prognosis-related genes in osteosarcoma patients. Based on the filtered genes, the TELscore for each sample was calculated using the following formula:

$$TELscore=\sum_{i=1}^{n}\text{Coe}f_{i}*X_{i},$$

where Coef_i_ represents the risk coefficient calculated by the lasso Cox model for each factor and X_i_ represents the expression value of gene. Next, the R packages survival and survminer and two-sided log rank tests were used to determine the value of TELscore, and patients were divided into low TELscore and high TELscore groups according to the median of TELscore.

### 2.3 Survival analysis

The “survival” and “survminer” packages in R were utilized to estimate the overall survival rates of various groups using the Kaplan–Meier method. The log-rank test was employed to assess the significance of differences in survival rates among these groups. Additionally, the R language’s pROC package (Robin et al., 2011) was used to generate the receiver operating characteristic (ROC) curve and calculate the AUC value. Furthermore, the timeROC package (Blanche et al., 2013) was utilized to generate the timeROC curve and compute its area under the curve (AUC) value.

### 2.4 Differentially expressed genes

Based on the function of the “limma” package (version 3.52.4) (Ritchie et al., 2015) in R language, the differentially expressed genes (DEGs) between low and high TELscore groups were screened using the criteria |log2FC| > 0.5 and FDR <0.05.

### 2.5 Functional enrichment and immune cell infiltration analyses

The DEGs were subsequently submitted for enrichment analysis using Gene Ontology (GO), which included biological processes, molecular functions, and cellular components, and the Kyoto Encyclopedia of Genes and Genomes (KEGG) using the “ClusterProfiler” function package (version 4.7.1.2) (Yu et al., 2012) in R language. The significantly enriched pathways were screened using the criteria P < 0.05.

The GSVA were performed using the c2.cp.kegg.v2023.1.Hs.symbols and c5.go.v2023.1.Hs.symbols gene sets from the Molecular Signature Database (https://www.gsea-msigdb.org/gsea/msigdb). The significantly enriched pathways were screened using the criteria |logFC| < 0.5 and P < 0.05.

The ssGSEA algorithm of the R package “GSVA” (version 1.46.0) (Charoentong et al., 2017) was used to calculate the abundance of 28 specific immune cell types. The stromal and immune cell scores of samples were calculated by the “estimate” function package (https://R-Forge.R-project.org/projects/estimate/).

### 2.6 Single-cell data analysis

Single-cell data processing was performed using the R language “Seurat” package (version 4.3.0). Single-cell data were filtered to remove low-quality single cells with characteristic gene counts less than 200 and containing greater than 5% mitochondrial count. The scRNA-seq data were normalized using the function “normalizeddata”. Principal component analysis (PCA) on the data was performed using the “RunPCA” function. Unsupervised clustering of major cell subtypes was performed using the findclusters function in Seurat and visualized using tSNE. Cell-type annotation was performed using the singleR software package and refined with manual annotation. DEGs between the two TELscore groups of osteosarcoma cells were calculated using “findmarkers” function. The “monocle” function package (version 2.26.0) was used for pseudotime analysis. The R package “Italk” (https://github.com/Coolgenome/iTALK, version 0.1.0) was applied to explore the cell communication atlas.

### 2.7 Drug sensitivity analysis

Drug sensitivity analysis was performed using the oncopredict package (Maeser et al., 2021) in R language. The GDSC2_Expr expression matrix, which contains data on 17,419 genes across 805 cell lines, along with the GDSC2_Res drug sensitivity dataset, which provides IC_50_ values for 198 drugs in these cell lines, was employed as the training set. The calcPhenotype function was applied to compute the predicted IC_50_ value for the sample based on its expression matrix.

### 2.8 Cell culture and transfection

The osteoblast cell line, hFOB1.19, was purchased from the Cell Bank of the Committee on Type Culture Collection of the Chinese Academy of Sciences (Shanghai, China). Two osteosarcoma cell lines (HOS and U-2OS) were purchased from the Procell Life Science & Technology Co., Ltd. (Wuhan, China), and osteosarcoma cell line, SAOS-2, was purchased from the iCell Bioscience Inc. (Shanghai, China). hFOB1.19 cells (Vero cells) were cultured in the DMEM (PM 150270, Procell, Wuhan, China) supplemented with 10% FBS and 1% PS at 37°C in 95% air/5% CO_2_. HOS cells were maintained in MEM (PM 150410, Procell, Wuhan, China) with 10% FBS and 1% P/S at 37 °C in 5% CO_2_ cell culture incubator. U-2OS and SAOS-2 cells were cultured in McCoy’s 5A medium (PM 150710, Procell, Wuhan, China) with 10% FBS and 1% P/S at 37°C in a humidified atmosphere of 5% CO_2_ and 95% air.

The SAOS-2 cells were transfected with OE-NC and OE-MAP7, respectively, using Lipofectamine 2000 C transfection reagent (AQ11669, Beijing Aoqing Biotechnology Co., Ltd., China) according to the manufacturer’s protocol.

### 2.9 qRT-PCR assay

TriQuick Reagent (R1100, Solarbio, China) was utilized to extract total RNA from the cells. Reverse transcription was carried out using the Evo M-MLV Reverse Transcription Reagent Master Mix (AG11706-S, Accurate Biology, Changsha, China). Subsequently, the qRT-PCR assay was performed using the SuperStar Universal SYBR Master Mix (CW3360M, JiangSu CoWin Biotech Co., Ltd., Jiangsu, China) on a real-time fluorescence quantitative PCR instrument. The primer sequences are listed in Table 1. The thermal cycling program was as follows: pre-denaturation at 95°C for 30 s, followed by 40 cycles of denaturation at 95°C for 10 s, and annealing/extension at 60°C for 30 s. GADPH was used as the internal reference. mRNA expression levels were quantified using the 2^−ΔΔCT^ method.

**TABLE 1:** Primer sequences for qPCR.

Genes	Forward primer (5′-3′)	Reverse primer (5′-3′)
MAP7	AAA​CTC​TTT​GTA​ACA​CCA​CCT​GA	GAT​GGA​GAT​ACA​GCC​CTT​CG
GAPDH	GAA​GGT​GAA​GGT​CGG​AGT​C	GAA​GAT​GGT​GAT​GGG​ATT​TC

### 2.10 Western blotting analysis

The protein was extracted using the RIPA buffer (R0010, Solarbio, Beijing, China) from cells. The lysates were centrifuged (at 12,000 rpm for 10 min at 4°C) to collect the supernatant. The protein was separated by SDS-PAGE and then transferred onto the PVDF membrane with a constant current of 250 mA. The membranes were blocked in 5% nonfat dry milk for 2 h and incubated with primary antibodies. Subsequently, the membranes were washed with 1×TBST and incubated for 1 h with a secondary antibody. The primary antibodies used in this study were MAP7 polyclonal antibody (1: 1,000, 13446-1-AP, Proteintech) and GAPDH monoclonal antibody (1:10000, 60004-1-Ig, Proteintech). The secondary antibody used in this study was horseradish enzyme-labeled goat anti-mouse IgG (H + L) (1:10,000, ZB-2301, Beijing Zhongshan Jinqiao Biotechnology Co., Ltd., China). Finally, the protein bands were detected using a fully automated chemiluminescence image analysis system (Chemi6000, Clinx, Shanghai, China).

### 2.11 Cell counting Kit-8 assay

The Cell Counting Kit-8 (CCK-8) assay was performed using the CCK-8 reagent (C0037, Beyotime Biotechnology). SAOS-2 cells transfected with OE-NC and OE-MAP7, along with control cells, were seeded into 96-well plates at a density of 5,000 cells per well. After 24, 48, and 72 h, the cells were incubated with the CCK-8 reagent at 37°C for 2 h. Subsequently, the absorbance of each well was measured at 450 nm using a microplate reader.

### 2.12 Cell invasion and migration

The Transwell assay (Costa, 3422) was utilized to assess the migration and invasion capabilities of the cells. A measure of 100 µL of the cell suspension (5 × 10^5^ cells/mL) was added to the top chamber, which was either coated with Matrigel (356234, Corning) or left uncoated. Subsequently, 600 µL of the culture medium supplemented with 20% serum was added to the lower chamber, and the cells were incubated for 48 h. After incubation, the cells were stained with a 0.1% crystal violet solution (Q/12GF, Tianjin Guangfu Chemical Industry Institute, China) for 1 h and then imaged using an inverted microscope (IMT-2, Olympus).

### 2.13 Statistical analysis

The Wilcoxon rank sum test was applied to compare gene expression differences among different groups. The single-sample Kolmogorov–Smirnov test was used to test whether the data conform to the normal distribution, and Pearson/Spearman correlation analysis was performed using the R language “cor” function. Differences were considered statistically significant when p < 0.05. R software version 4.2.2 was used for all of the aforementioned statistical studies.

Moreover, all experimental data were statistically analyzed using GraphPad Prism 9.5.0 software and were expressed as the mean ± standard deviation (SD). Each experiment was repeated at least three times with three replications per experiment. The unpaired two-tailed t-test was used for comparison between two groups, and the difference was statistically significant when p < 0.05.

## 3 Results

### 3.1 Construction of the telomere-related signature in osteosarcoma

**FIGURE 1:**
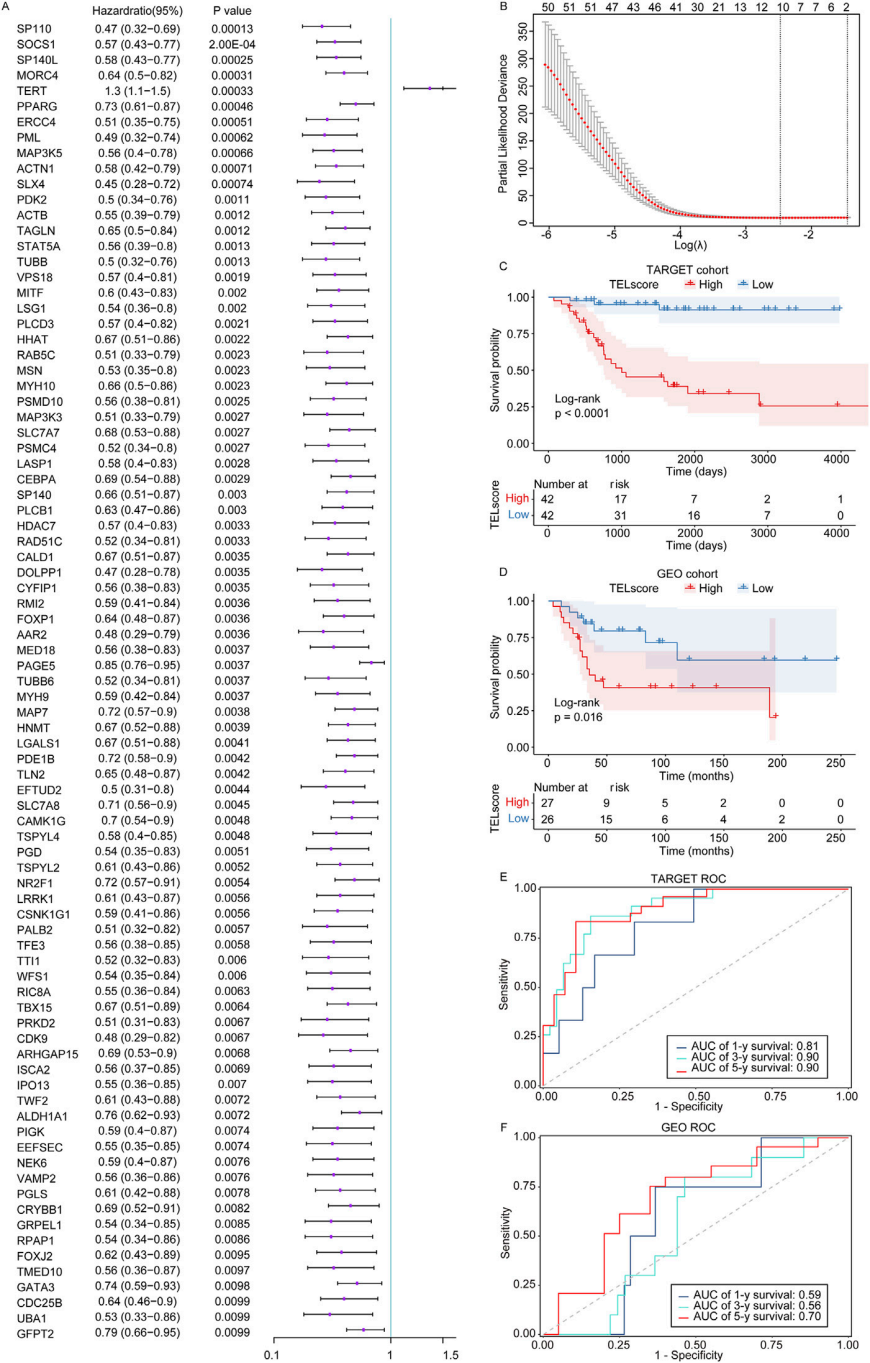
Construction of the telomere-related signature in osteosarcoma. **(A)** The univariate Cox regression analysis was performed on the telomere-related genes in osteosarcoma. Hazard ratio with 95% confidence interval. **(B)** The LASSO regression analysis was performed on the prognostic telomere-related genes in osteosarcoma. **(C, D)** Kaplan–Meier curves of the high and low TELscore groups in the TARGET cohort and GSE21257 dataset. **(E, F)** 1-year, 3-year, and 5-year ROC curves of the telomere-related signature in the TARGET cohort and GSE21257 dataset.

First, in the training set TARGET cohort (including 84 osteosarcoma samples with complete survival information), we performed the univariate Cox regression analysis using the 2086 telomere-related genes as continuous variables and calculated the hazard ratio (HR) of each gene. Among these, 85 telomere-related genes were found to be significantly associated with the prognosis of osteosarcoma patients (Figure 1A, P < 0.01). Furthermore, LASSO Cox regression analysis identified 10 genes (SP110, HHAT, TUBB, MORC4, TERT, PPARG, MAP3K5, PAGE5, MAP7, and CAMK1G) that exhibited a remarkable correlation with the prognosis of osteosarcoma patients (Figure 1B, minimum lambda value).

Subsequently, we weighted the expression of these 10 genes with the regression coefficients from the LASSO Cox regression analysis and established telomere-related signature to predict the survival of patients. TELscore = (−0.28285616) × express value of SP110 + (−0.01519659) × express value of HHAT + (−0.04605673) × express value of TUBB + (−0.19292665) × express value of MORC4+0.13670374 × express value of TERT + (−0.03302422) × express value of PPARG + (−0.05545063) × express value of MAP3K5 + (−0.05689784) × express value of PAGE5 + (−0.04040167) × express value of MAP7 + (−0.01099716) × express value of CAMK1G. In the TARGET cohort, the high TELscore group was linked to inferior prognosis of osteosarcoma patients compared to the low TELscore group (Figure 1C), which was also found in the GSE21257 dataset (Figure 1D). Moreover, the ROC analysis showed that the AUC values of 1-, 3-, and 5-year overall survival in the training set TARGET cohort were 0.81, 0.90, and 0.90, respectively (Figure 1E). In the validation set GSE21257, the AUC values of 1-, 3-, and 5-year overall survival in the training set TARGET cohort were 0.59, 0.56, and 0.70, respectively (Figure 1F). These findings suggested that the telomere-related signature could effectively predict the prognosis of osteosarcoma patients in the TARGET cohort.

### 3.2 Prognostic value of the telomere-related signature

**FIGURE 2:**
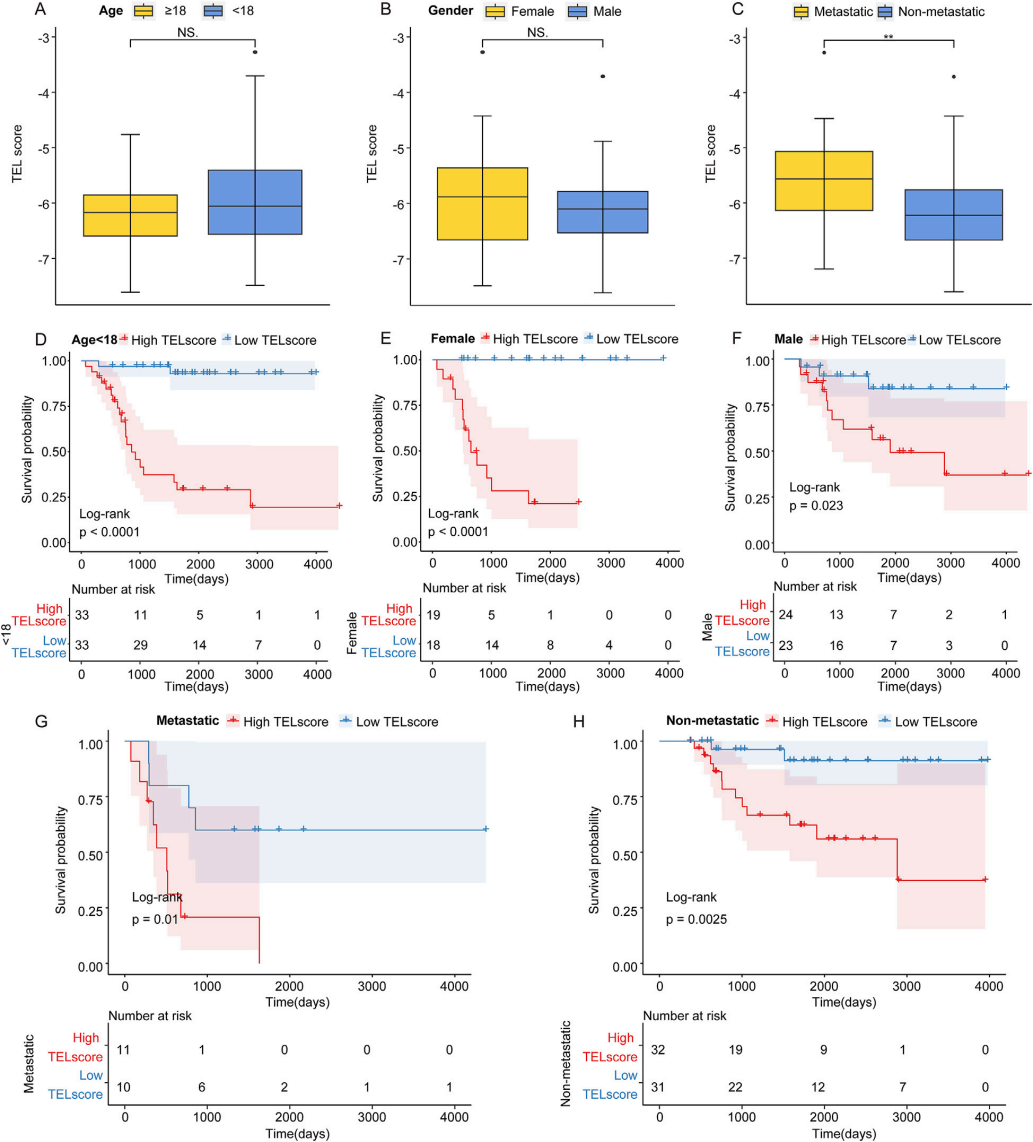
Prognostic value of the telomere-related signature. **(A)** TELscore levels in osteosarcoma patients with age ≧18 and <18 years. **(B)** Level of TELscore in female and male osteosarcoma patients. **(C)** Level of TELscore in metastatic and non-metastatic osteosarcoma patients. **(D)** Kaplan–Meier curves of the high and low TELscore groups in osteosarcoma patients with age <18. **(E, F)** Kaplan–Meier curves of the high and low TELscore groups in the female and male osteosarcoma patients. **(G, H)** Kaplan–Meier curves of the high and low TELscore groups in metastatic and non-metastatic osteosarcoma patients.

In the TARGET cohort, we discovered that the TELscore was not significantly differential between the osteosarcoma patients with age ≧18 and osteosarcoma patients <18 years and female and male osteosarcoma patients (Figures 2A, B). Compared to non-metastatic osteosarcoma samples, the TELscore was remarkably increased in metastatic samples (Figure 2C). Moreover, we found that in osteosarcoma patients with age <18 years, the high TELscore was related to worse prognosis of patients (Figure 2D). In the female and male groups, the osteosarcoma patients in the high TELscore group exhibited inferior prognosis (Figures 2E, F). In metastatic and non-metastatic osteosarcoma patients, the high TELscore group was linked to a poor prognosis (Figures 2G, H). These findings suggested that the TELscore was related to metastasis and inferior prognosis of osteosarcoma.

### 3.3 Functional annotation of the telomere-related signature

**FIGURE 3:**
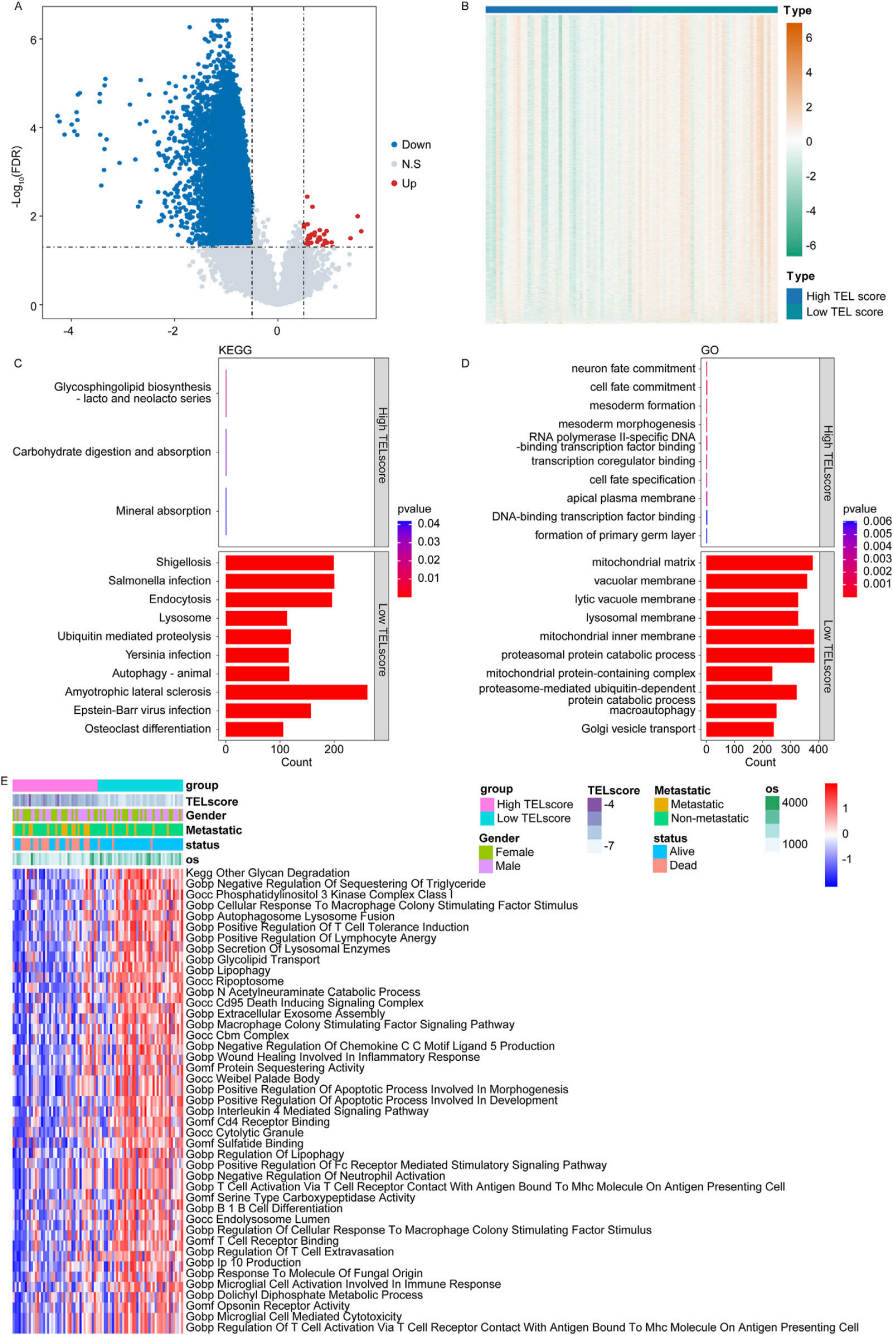
Functional annotation of the telomere-related signature. **(A)** Volcano plot for the DEGs between the high and low TELscore groups. **(B)** Heatmap for the DEGs between the high and TELscore groups. **(C)** KEGG enrichment analysis for differentially expressed genes between the high and low TELscore groups. **(D)** GO enrichment analysis for the differentially expressed genes between the high and low TELscore groups. **(E)** The GSVA for the differentially expressed genes between the high TELscore and low TELscore groups.

In the TARGET cohort, we identified a total of 11,523 DEGs between the high and low TELscore groups, including 43 upregulated genes and 11,480 downregulated genes (Figures 3A, B, high vs. low). The KEGG enrichment analysis revealed that the glycosphingolipid biosynthesis-lacto and neolacto series, carbohydrate digestion and absorption, and mineral absorption were significantly enriched in the high TELscore group, while a total of 158 signaling pathways were notably enriched in the low TELscore group (Figure 3C; Supplementary Table S2). The GO enrichment analysis indicated that a total of 168 signaling pathways were markedly enriched in the high TELscore group, whereas 2,829 signaling pathways were dramatically enriched in the low TELscore group (Figure 3D; Supplementary Table S2). Moreover, GSVA demonstrated that the scores of 43 signaling pathways were significantly different between the high and low TELscore groups (Figure 3E; Supplementary Table S2).

### 3.4 Predictive value of the telomere-related signature in the immune microenvironment of osteosarcoma

**FIGURE 4:**
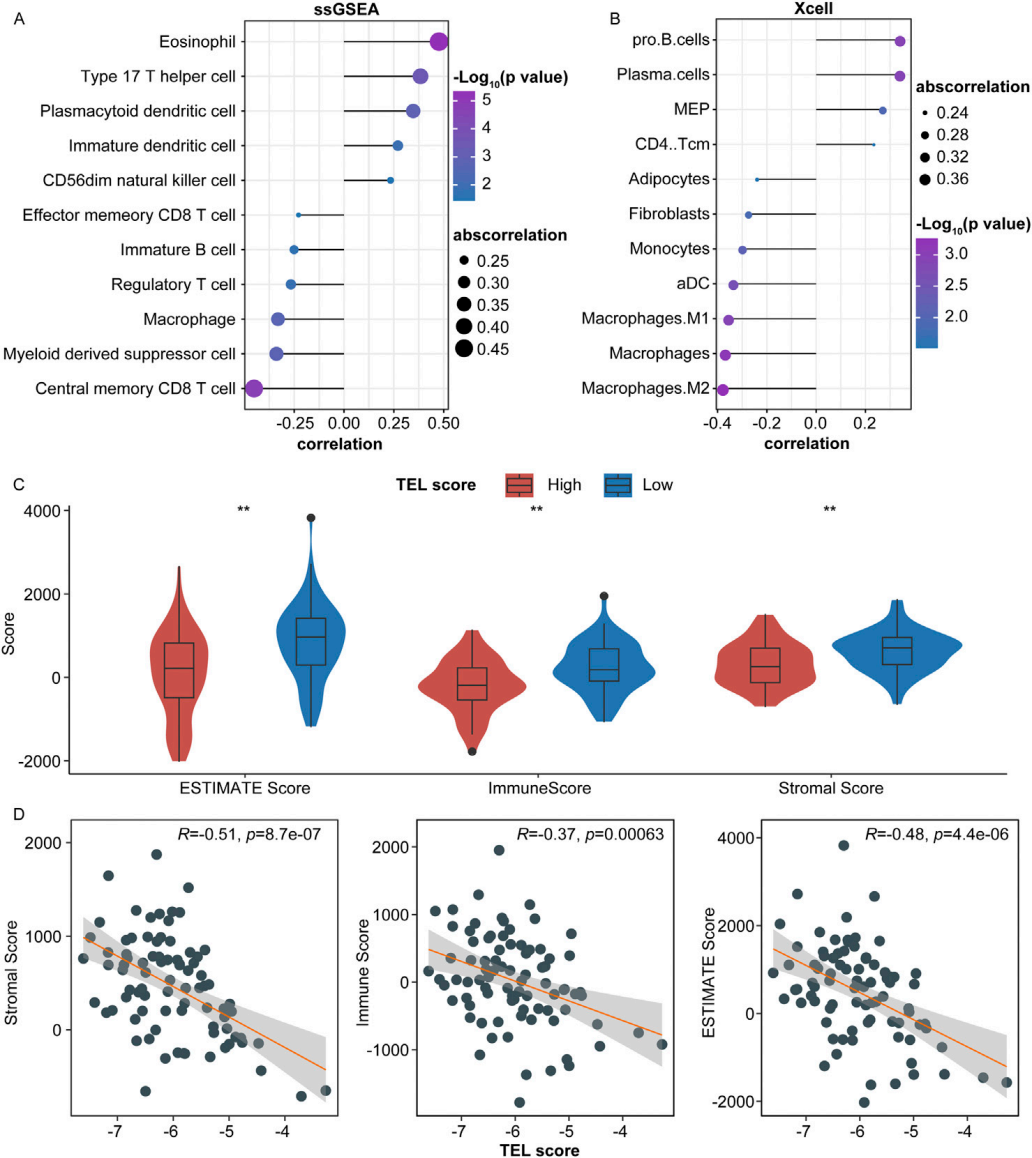
Predictive value of the telomere-related signature in the immune microenvironment of osteosarcoma. **(A)** Correlation of TELscore with the abundance of immune cells. **(B)** Correlation of TELscore with immune cell infiltration. **(C)** ESTIMATE score, immune score, stromal score levels in the high and low TELscore groups. **(D)** Correlation between TELscore and stromal score, immune score, and ESTIMATE score.

In the TARGET cohort, we calculated the correlation between TELscore and the abundance of 28 specific immune cell types. Our analysis revealed that TELscore exhibited a significantly negative correlation with six immune cells, including central memory CD8+T cells and myeloid-derived suppressor cells. Conversely, TELscore showed a notably positive association with five immune cells, such as eosinophils and type 17 T helper cells (Figure 4A, P < 0.05). Additionally, in the Xcell database (https://xcell.ucsf.edu/), TELscore was positively correlated with the relative proportions of four immune cell infiltrations (CD4 Tcm, MEP, plasma cells, and Pro B cells) and negatively associated with the relative proportions of seven immune cell infiltrations, including M2 macrophages and macrophages (Figure 4B; Supplementary Table S3).

Furthermore, we calculated the immune scores for high and low TELscore groups within the TARGET cohort. The ESTIMATE score, immune score, and stromal score levels were lower in the high TELscore group than in the low TELscore group (Figure 4C). Moreover, TELscore was negatively correlated with the ESTIMATE, immune, and stromal scores (Figure 4D).

### 3.5 Single-cell sequencing analysis for the telomere-related signature in osteosarcoma

**FIGURE 5:**
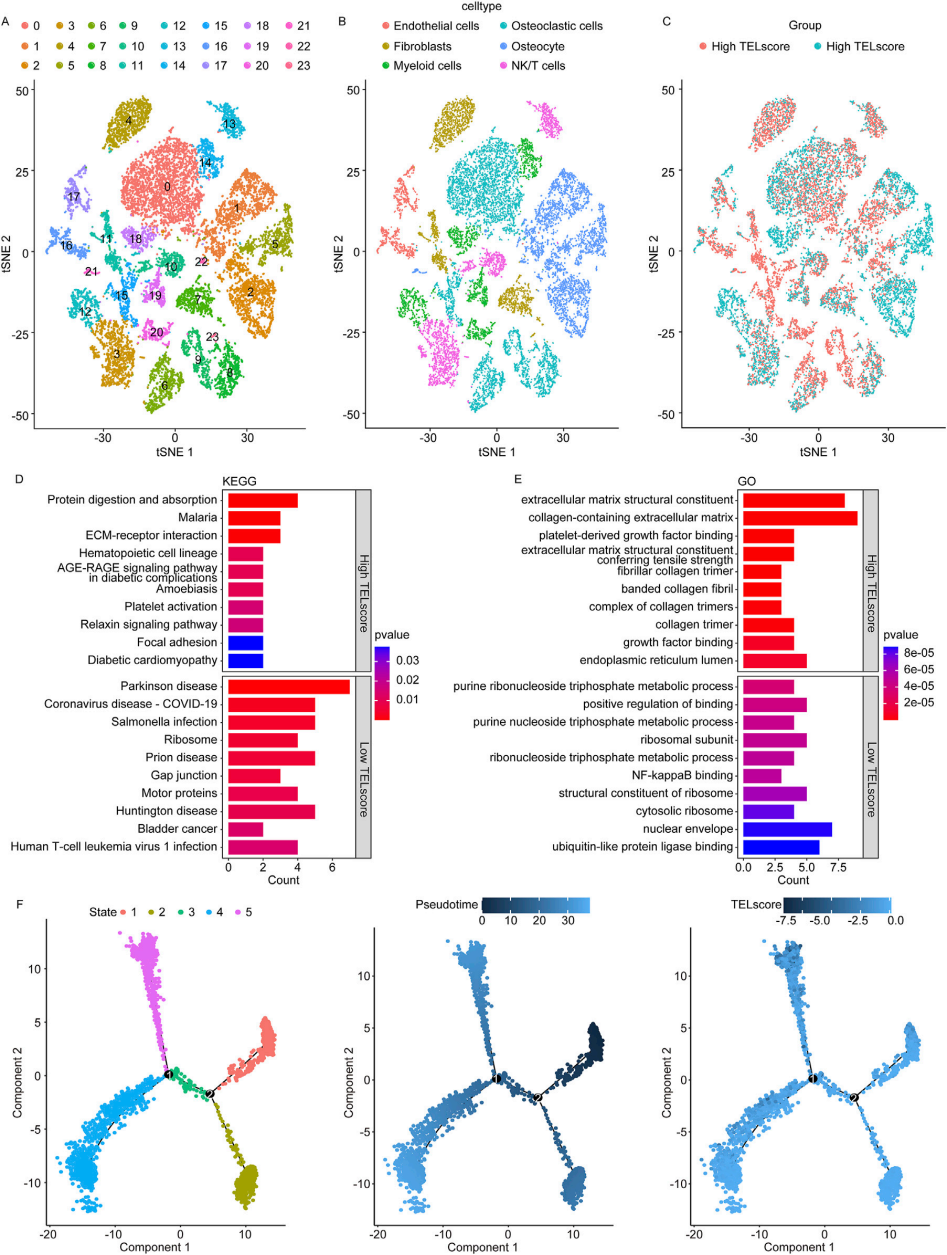
Single-cell sequencing analysis for the telomere-related signature in osteosarcoma. **(A)** Identified 24 cell clusters using Seurat. **(B)** Result of cell annotation. **(C)** Levels of the telomere-related signature score in identified cells. **(D)** Dot plot of KEGG enrichment analysis results of the high and low TELscore groups. **(E)** Dot plot of GO enrichment analysis results of the high and low TELscore groups. **(F)** Pseudotime analysis of different cell states on osteosarcoma cells (left), pseudotime mode of pseudotime analysis in osteosarcoma cells (middle), and pseudotime analysis of osteosarcoma cells by telomere-related signature score (right).

We obtained a gene expression map from the single-cell sequencing dataset GSE152048. We performed PCA to reduce dimensionality using 5,000 variable genes and identified 24 cell clusters using Seurat (Figure 5A). The results of cell annotation are presented in Figure 5B. The telomere-related signature score levels in the identified cells are presented in Figure 5C. We identified DEGs between the high and low TELscore groups in the GSE152048 dataset and performed the GO and KEGG enrichment analyses to obtain more function information. The KEGG enrichment analysis showed that 11 and 23 signaling pathways were remarkably enriched in the high and low TELscore groups, respectively (Figure 5D; Supplementary Table S4). The GO enrichment analysis showed that 233 and 429 signaling pathways were observably enriched in the high and low TELscore group, respectively (Figure 5E; Supplementary Table S4). Furthermore, we performed pseudotime analysis on osteosarcoma cells and identified five cell states (Figure 5F, left). As pseudotime increased (Figure 5F, middle), the osteosarcoma cells exhibited a tendency to increase TELscore (Figure 5F, right).

### 3.6 Cell communication pattern of the telomere-related signature

**FIGURE 6:**
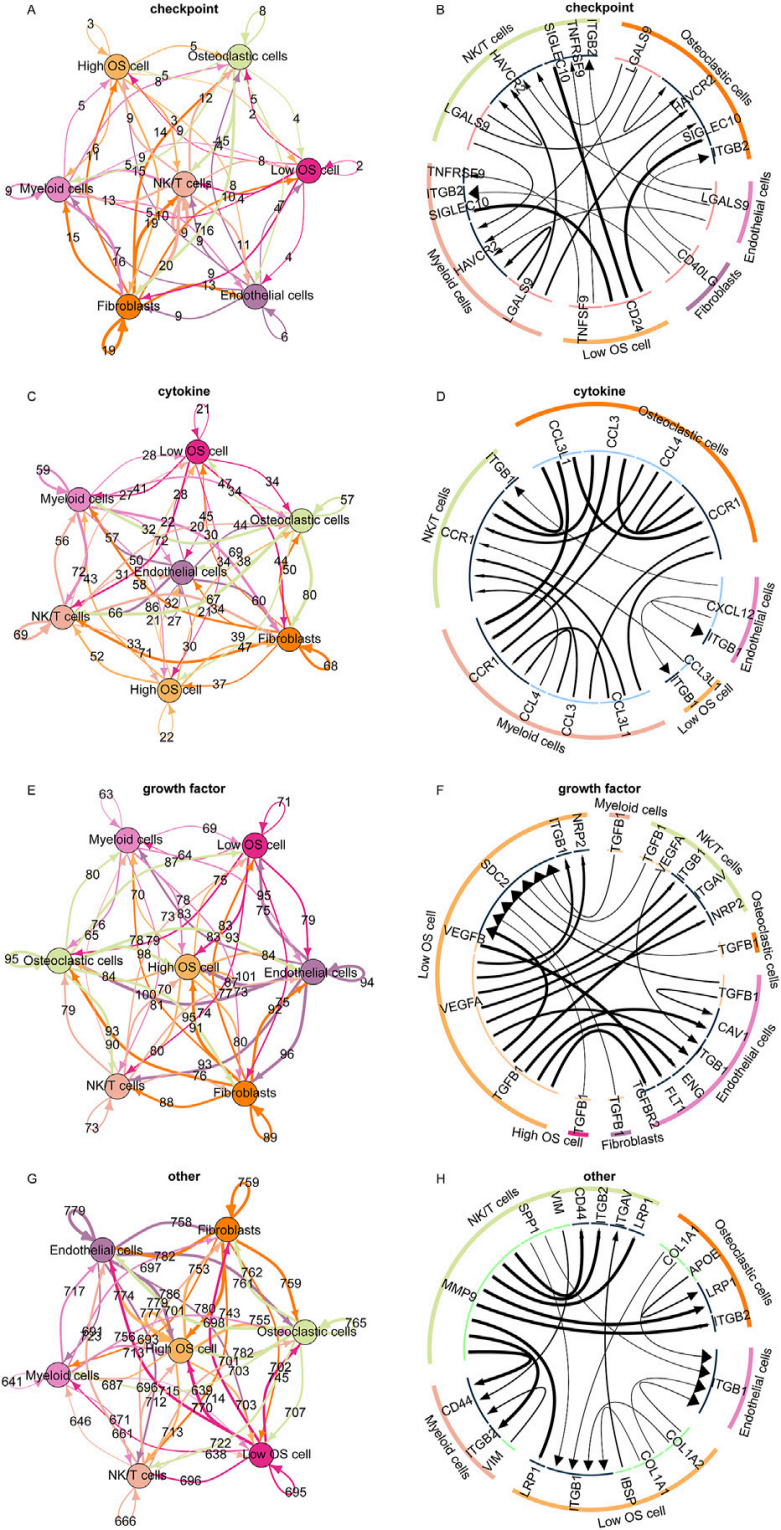
Cell communication pattern of the telomere-related signature. **(A, B)** Different cellular signaling pathways regarding checkpoints between the high and low TELscore groups of osteosarcoma cells and microenvironment cells. **(C, D)** Different cellular signaling pathways of cytokines between the high and low TELscore groups of osteosarcoma cells and microenvironment cells. **(E, F)** Different cellular signaling pathways of growth factors between the high and low TELscore groups of osteosarcoma cells and microenvironment cells. **(G, H)** Different cellular signaling pathways of others between the high and low TELscore groups of osteosarcoma cells and microenvironment cells.

Subsequently, we analyzed the different cellular signaling pathways of checkpoints, cytokines, growth factors, and others between the high and low TELscore groups of osteosarcoma cells and microenvironment cells. In the low TELscore group, TNFSF9 and CD24 were the most active signaling pathways of checkpoints in osteosarcoma cells (Figures 6A, B). In the low TELscore group, ITGB1 and CCL3L1 were the most active signaling pathways of cytokines in osteosarcoma cells (Figures 6C, D). In the high TELscore group, TGFB was the most active signaling pathway of growth factors in osteosarcoma cells (Figures 6E, F). In the high TELscore group, LRP1, ITGB1, IBSP, COL1A1, and COL1A2 were the most active signaling pathways of others in osteosarcoma cells (Figures 6G, H).

### 3.7 Predictive value of telomere-related signature in the drug sensitivity of osteosarcoma

**FIGURE 7:**
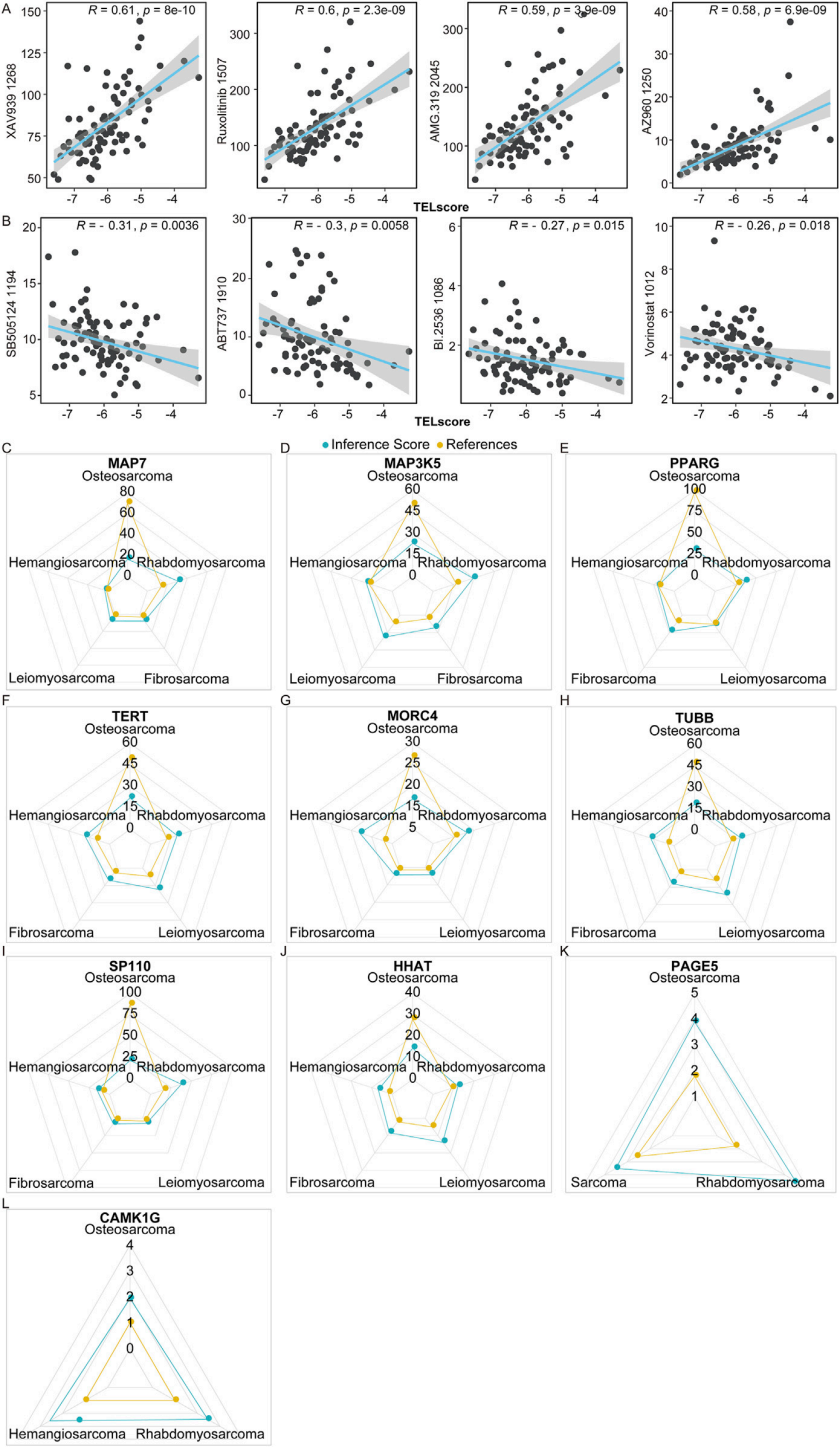
Predictive value of telomere-related signature in the treatment response of osteosarcoma. **(A,B)** Correlation of TELscore with drugs. **(C–L)** MAP7, MAP3K5, PPARG, TERT, MORC4, TUBB, SP110, and HHAT exhibited strong values for the development and occurrence of osteosarcoma.

In the TARGET cohort, we analyzed the correlation between TELscore and drug sensitivity using R language “oncopredict” (version 0.2). The TELscore exhibited a prominent positive correlation with the IC_50_ values of 154 drugs, such as AZ960_1250, AMG.319_2045, Ruxolitinib_1507, and XAV939_1268 (Figure 7A). Conversely, it demonstrated a significant negative association with the IC_50_ values of SB505124_1194, ABT737_1910, BI.2536_1086, and Vorinostat_1012 (Figure 7B; Supplementary Table S5, P < 0.05). Additionally, we analyzed the inference score of 10 genes using the CTD database (http://ctdbase.org/). All 10 genes were identified as targets for osteosarcoma (Figures 7C–L), with MAP7, MAP3K5, PPARG, TERT, MORC4, TUBB, SP110, and HHAT demonstrating significant relevance to the development and progression of osteosarcoma.

### 3.8 Low MAP7 expression was correlated with a poor prognosis of osteosarcoma patients

**FIGURE 8:**
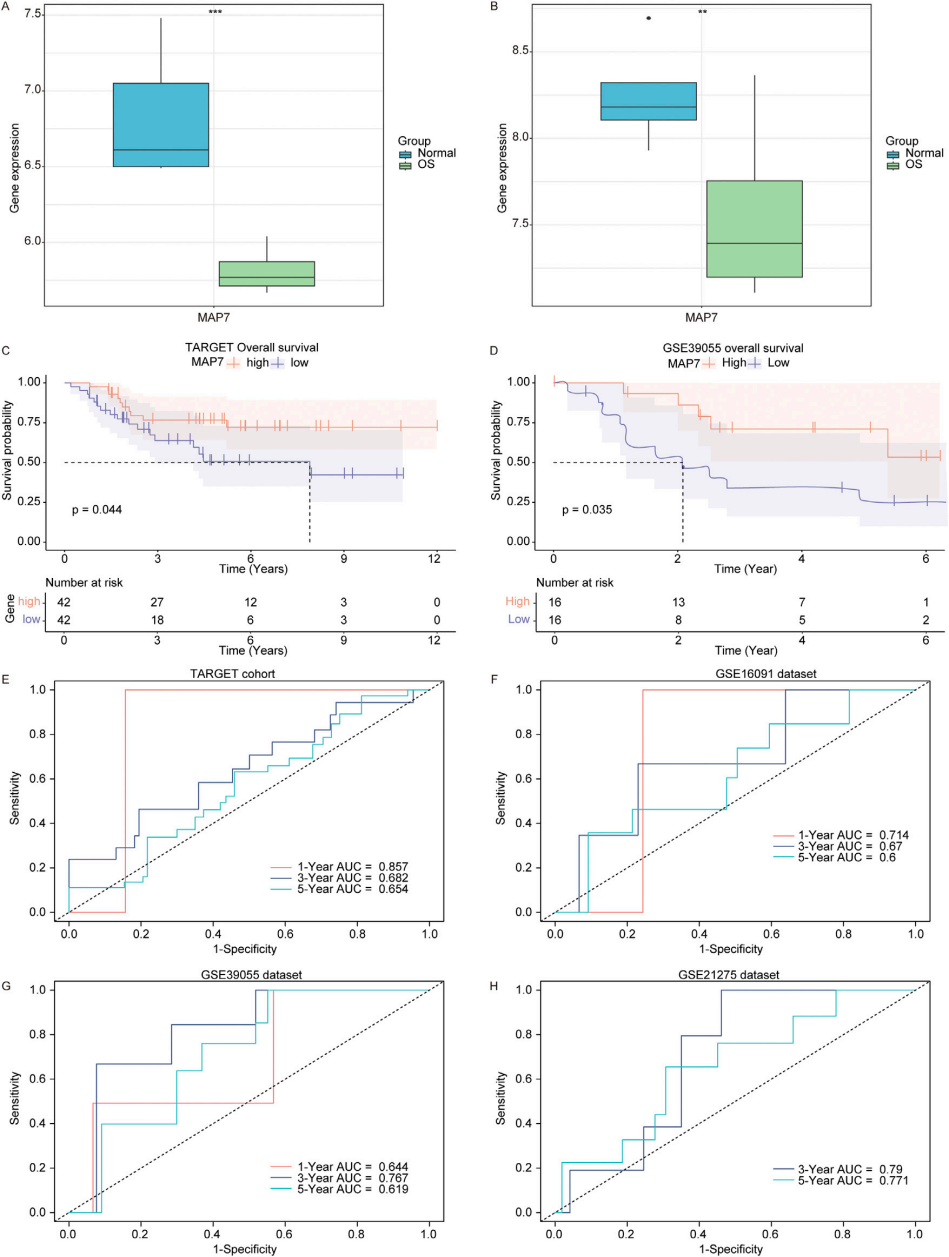
Low MAP7 expression was correlated with a poor prognosis of osteosarcoma patients. Expression of MAP7 in osteosarcoma and normal samples in the GSE16088 **(A)** and GSE28424 **(B)** datasets. KM survival curve (high MAP7 expression vs. low MAP7 expression patients) for overall survival in the TARGET **(C)** and GSE39055 **(D)** cohort. Time-dependent AUC for MAP7 in the TARGET cohort **(E)**, GSE16091 dataset **(F)**, GSE39055 **(G)** dataset, and GSE21275 **(H)** dataset. ** means P < 0.01 and *** means P < 0.001.

Subsequently, we analyzed the prognostic role of MAP7 in osteosarcoma patients and found that the expression of MAP7 was downregulated in osteosarcoma samples compared to normal samples in the GSE16088 (Figure 8A) and GSE28424 datasets (Figure 8B). In the TARGET and GSE39055 cohort, we divided osteosarcoma patients into MAP7^high^ and MAP7^low^ groups according to the median expression value of MAP7. The Kaplan–Meier survival analysis showed that the MAP7^low^ group had a worse prognosis than the MAP7^high^ groups in the TARGET (Figure 8C) and GSE39055 cohorts (Figure 8D). Furthermore, we discovered that the AUC values of MAP7 at 1, 3, and 5 years were 0.857, 0.682, and 0.654 in the TARGET cohort, respectively (Figure 8E). The AUC values in the GSE16091 cohort were 0.714, 0.67, and 0.6 for 1, 3, and 5 years, respectively (Figure 8F). In the GSE39055 cohort, the AUC values were 0.644 (1 year), 0.767 (3 years), and 0.619 (5 years) (Figure 8G). In the GSE21275 cohort, the AUC values were 0.790 (3 years) and 0.771 (5 years) (Figure 8H). These results suggested that MAP7 was closely correlated with the prognosis of osteosarcoma patients.

### 3.9 Overexpression of MAP7 might inhibit tumor proliferation, migration, and the invasion of osteosarcoma cells

**FIGURE 9:**
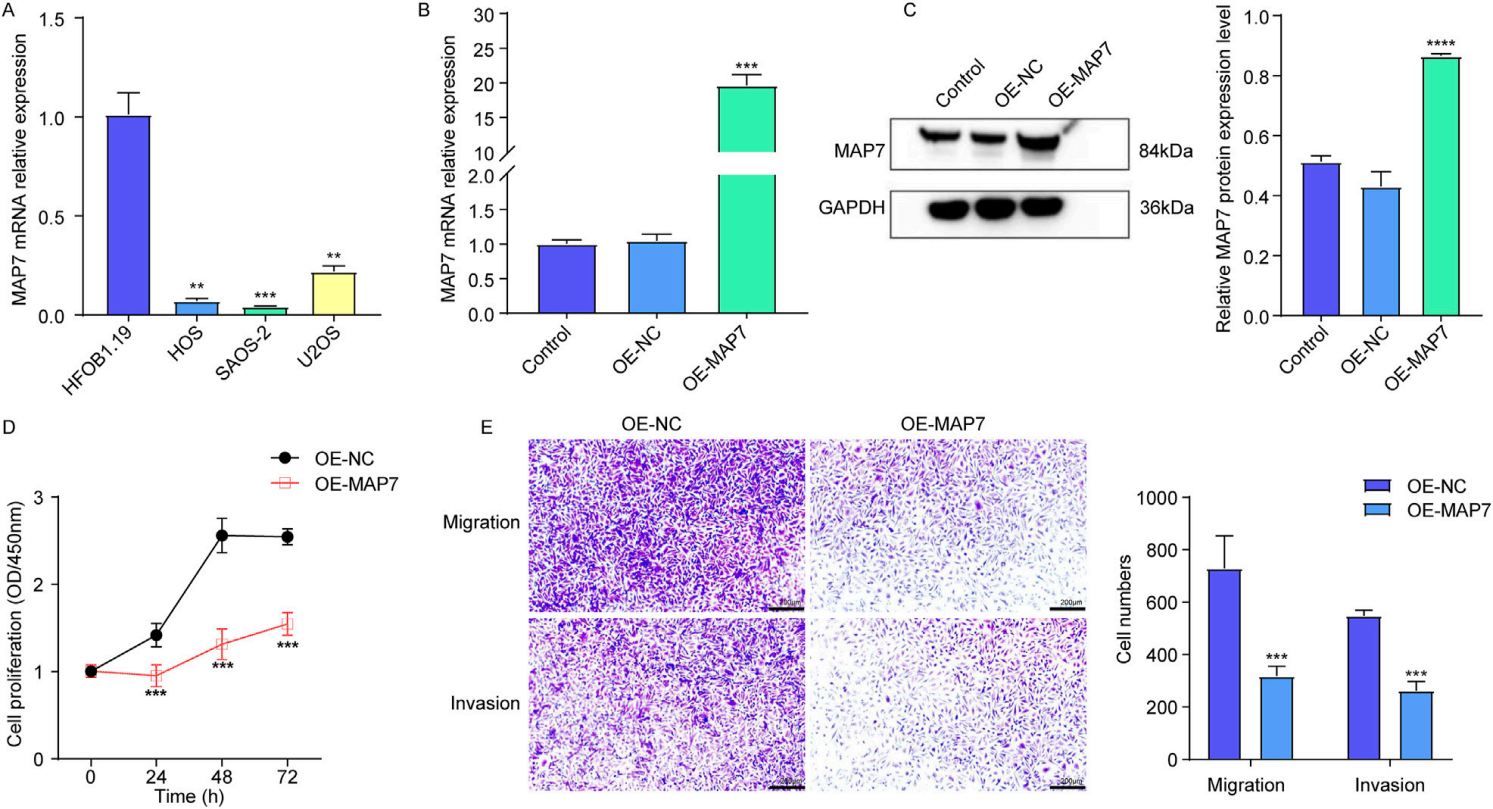
Overexpression of MAP7 might inhibit tumor proliferation, migration, and the invasion of osteosarcoma cells. **(A)** Expression of MAP7 in hFOB1.19, HOS, U-2OS, and SAOS-2 cells. Overexpression efficiency of MAP7 was evaluated by qRT-PCR **(B)** and Western blotting **(C)**. **(D)** Cell proliferation was detected using CCK-8 assays. **(E)** The ability of cell migration and invasion was detected using the Transwell assay. * means P < 0.05, **P < 0.01, *** means P < 0.001, and **** means P < 0.0001.

Finally, to explore the role of MAP7 in the progression of osteosarcoma, we first confirmed the expression of MAP7 mRNA in several osteosarcoma cell lines. Compared to hFOB1.19 cells, the level of MAP7 mRNA expression was significantly decreased in HOS, U-2OS, and SAOS-2 cells (Figure 9A). Furthermore, we constructed the MAP7 overexpression model in SAOS-2 cells and found that MAP7 expression was remarkably increased in the OE-MAP7 group compared to the ON-NC group (Figures 9B, C). Moreover, the overexpression of MAP7 significantly reduced cell proliferation (Figure 9D) and the ability of cell migration and invasion in SAOS-2 cells (Figure 9E). These findings suggested that the overexpression of MAP7 might inhibit tumor proliferation, migration, and the invasion of osteosarcoma cells.

## 4 Discussion

Numerous studies have demonstrated the pivotal role of telomeres in the intricate processes of cancer development and progression. It has been reported that telomere shortening may act as a tumor suppressor by inhibiting cell growth, while also leading to widespread genomic instability, which promotes cancer development (Okamoto and Seimiya, 2019; Li S. C. et al., 2022). Therefore, a comprehensive understanding of telomeres in cancer is essential for the development of innovative and effective therapeutic strategies. Although many studies have focused on telomere length in cancer and its implications for osteosarcoma prognosis, there has been a lack of comprehensive examination regarding the involvement of telomere-related genes in the prognosis of osteosarcoma.

In the present study, we developed a telomere-related signature for osteosarcoma patients based on 10 telomere-related genes (SP110, HHAT, TUBB, MORC4, TERT, PPARG, MAP3K5, PAGE5, MAP7, and CAMK1G). The TUBB protein was found to be downregulated in osteosarcoma samples (Li et al., 2010). TERT was highly expressed in cisplatin-resistant osteosarcoma cells, where it shuttles from the nucleus to the mitochondrion in response to cisplatin therapy, thereby suppressing cisplatin-induced apoptosis in osteosarcoma cells. This mechanism may be particularly crucial in combating drug resistance (Zhang et al., 2017). Xie et al. (2023) indicated that the knockdown of TERT could decrease proliferation and inhibit the invasion and migration of osteosarcoma 143B and U2OS cells. PPARG has been identified as a hub gene in the onset of osteosarcoma (Li L. et al., 2022), and the knockout of PPARG significantly represses the growth of osteosarcoma cells (Yuan et al., 2023). The expression of MAP3K5 was reported to be inversely correlated with the survival risk of osteosarcoma (Li et al., 2020). Compared to normal human osteoblast hFOB1.19 cells, MAP3K5 expression was significantly decreased in osteosarcoma cells, and its knockdown promoted the migration of these cells (Yang et al., 2023). Accordingly, in osteosarcoma, TUBB and MAP3K5 were believed to function as tumor suppressors, actively inhibiting tumor growth and progression, while TERT and PPARG appear to act as cancer promoters, exacerbating the development of osteosarcoma.

Although the roles of SP110, HHAT, MORC4, PAGE5, MAP7, and CAMK1G in osteosarcoma have rarely been reported, their involvement in other types of cancer has been revealed. In hepatitis B virus-induced hepatocellular carcinoma, high SP110 expression was correlated with a shorter survival rate among patients (Sengupta et al., 2022). HHAT knockdown suppresses Shh pathway activation and the development of non-small cell lung cancer cells *in vitro*, as well as tumor growth *in vivo* in mice xenografts (Rodriguez-Blanco et al., 2013). MORC4 was found to be overexpressed in breast cancer tissues, and its downregulation by miR-193b-3p influences the proliferation of breast cancer cell via regulating apoptosis (Yang et al., 2019). PAGE5 was reported to act as a mediator of chemoresistance in human small cell lung cancer (Wollenzien et al., 2023). CAMK1G has been identified as a significant predictor of favorable overall survival of patients with prostate adenocarcinoma (Zhao et al., 2020). MAP7 has been shown to be highly in breast cancer and cervical cancer, where it enhances the invasion and migration of breast cancer and cervical cancer cells (Zhang et al., 2020; Wang et al., 2022; Wang et al., 2023). MAP7 (ensconsin, E-MAP-115) is a ubiquitous microtubule-associated protein that organizes the microtubule cytoskeleton in mitosis and neuronal branching (Chaudhary et al., 2019). It has been reported that the dysregulation of microtubule dynamics is associated with cancer development (Tala et al., 2014; Verma et al., 2023), suggesting that microtubule-targeting drugs are the basis of cancer chemotherapy. Lin et al. (2020) have demonstrated that the high expression of abnormal spindle microtubule assembly in osteosarcoma promotes the proliferation of osteosarcoma cells. Thus, we hypothesized that MAP7 might also play a critical role in the progression of osteosarcoma. Accordingly, to investigate the role of MAP7 in osteosarcoma progression, we established an MAP7-overexpressing osteosarcoma cell model in the SAOS-2 cell. We discovered that MAP7 expression was significantly reduced in osteosarcoma cells, and low expression of MAP7 was associated with poor prognosis of osteosarcoma patients. Moreover, the overexpression of MAP7 significantly reduced cell proliferation, the ability of cell migration, and invasion in osteosarcoma cells. These results indicated that MAP7 might play an important role in the progression and prognosis of osteosarcoma.

In both the training and validation sets, the high TELscore group was correlated with a poor prognosis of osteosarcoma patients. The AUCs for 1-, 3-, and 5-year overall survival in the training set TARGET cohort were 0.81, 0.90, and 0.90, respectively. The development of osteosarcoma has been observed to correlate with the age and gender of patients (Sadykova et al., 2020; Lee et al., 2021). Therefore, we analyzed the correlation of telomere-related signature with age and gender. We discovered that the TELscore was not significantly differential between the osteosarcoma patients with age ≧18 and osteosarcoma patients <18 years and female and male osteosarcoma patients. In addition, osteosarcoma has the property of systemic metastasis, especially pulmonary metastasis. Pulmonary metastasis remains the primary cause of death in osteosarcoma cases, and over 90% of patients succumb to the effects of these lung metastases (Weeden et al., 2001; Kim et al., 2010). Accordingly, we explored the relationship between telomere-related signature and metastasis of osteosarcoma. The TELscore was remarkably increased in metastatic osteosarcoma patients compared to non-metastatic patients. Furthermore, in osteosarcoma patients with age <18 years, female and male patients, metastatic and non-metastatic osteosarcoma patients, the high TELscore was correlated with worse prognosis. These findings indicated that telomere-related signature was not related to age and gender and was correlated with the metastasis of osteosarcoma patients, and telomere-related signature could effectively predict the prognosis of osteosarcoma patients.

The immune microenvironment in osteosarcoma represents a highly intricate system, characterized by its complexity, remarkable plasticity, and a close association with immune escape, uncontrolled cell proliferation, and the metastasis of osteosarcoma cells (Tian et al., 2023). Dendritic cells (DCs), which are common antigen-presenting cells (APCs) originating from the bone marrow (Collin and Ginhoux, 2019), play a crucial role as effective antigen-presenting cells capable of stimulating immature T cells and generating specific immunological responses (Geissmann et al., 2010). DCs are present in all forms of bone sarcomas, and their quantity generally correlates with the extent of tumor-associated macrophage (TAM) invasion. In chondrosarcoma and chordoma, DCs are either absent or present in low numbers (Inagaki et al., 2016). In osteosarcoma, the infiltration of DCs is closely associated with autophagy (Zhang et al., 2021). Furthermore, osteosarcoma cells can develop variations that tolerate DCs and phagocytosis during the progression of the disease, leading to reduced DC activation and, ultimately, immune escape (Le et al., 2021). The infiltration of T lymphocytes and macrophages plays a significant role in osteosarcoma immunotherapy. In a study involving 16 primary osteosarcoma patients, infiltrating T lymphocytes were identified in biopsy tumor tissue and peripheral blood samples; a higher level of positive T cells observed in tumor tissue compared to blood circulation (Han et al., 2016). It has been demonstrated that within the tumor microenvironment, TAMs can interact with other immune cells to participate in local inflammatory regulation and contribute to drug resistance in osteosarcoma (Han et al., 2019).

In the present study, the TELscore was positively associated with plasmacytoid dendritic cells and Type 17 T helper cells and was negatively correlated with central memory CD8+T cells, M2 macrophages, and regulatory T cells. Furthermore, TELscore was negatively correlated with the ESTIMATE, immune, and stromal scores. Accordingly, the telomere-related signature could regulate the immune microenvironment to affect the occurrence and progression of osteosarcoma. Moreover, we also found that the TELscore was positively correlated with the IC_50_ values of 154 drugs and negatively associated with four drugs. Considering the role of drug sensitivity in immunotherapy, we suggest that telomere-related signatures may shed new light on optimizing immunotherapeutic strategies for osteosarcoma.

A telomere-related signature was constructed for osteosarcoma patients based on SP110, HHAT, TUBB, MORC4, TERT, PPARG, MAP3K5, PAGE5, MAP7, and CAMK1G. The telomere-related signature had predictive values in the prognosis, immune microenvironment, and drug sensitivity of osteosarcoma. Furthermore, MAP7 might be a prognostic marker for osteosarcoma patients.

## Data Availability

The original contributions presented in the study are included in the article/Supplementary Material; further inquiries can be directed to the corresponding author.
