# Corrigendum: A telomere-related signature for predicting prognosis and assessing immune microenvironment in osteosarcoma

**DOI:** 10.3389/fphar.2025.1600217

**Published:** 2025-06-12

**Authors:** Shihao Li, Lina Zhang, Haiyang Zhang

**Affiliations:** ^1^ Department of Orthopedics, Zibo Central Hospital West Campus, Zibo, China; ^2^ Department of Hand and Foot Surgery, Zibo Central Hospital, Zibo, China

**Keywords:** osteosarcoma, telomere-related signature, prognosis, immune microenvironment, drug sensitivity, MAP7

In the published article, there was an error in Figure 9 as published. The micrograph scale was omitted in Figure 9E, meaning that both the scale bar and duplication needed correction. As such, we magnified the ruler to make the Transwell analysis images clearer. The corrected Figure 9 and its caption appear below.

**FIGURE 9:**
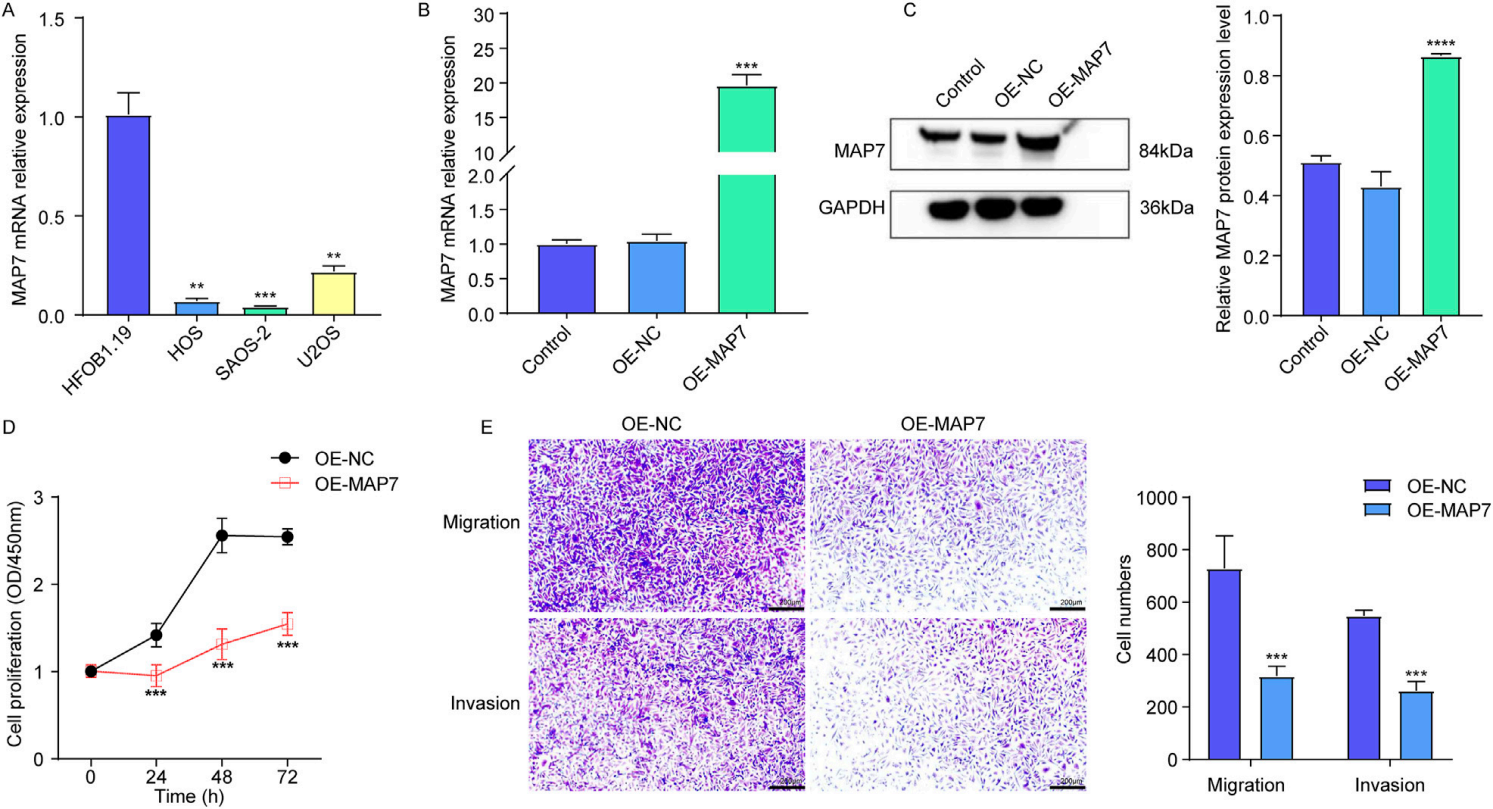
Overexpression of MAP7 might inhibit tumor proliferation, migration, and the invasion of osteosarcoma cells. **(A)** Expression of MAP7 in hFOB1.19, HOS, U-2OS, and SAOS-2 cells. Overexpression efficiency of MAP7 was evaluated by qRT-PCR **(B)** and Western blotting **(C)**. **(D)** Cell proliferation was detected using CCK-8 assays. **(E)** The ability of cell migration and invasion was detected using the Transwell assay. * means P < 0.05, **P < 0.01, *** means P < 0.001, and **** means P < 0.0001.

The authors apologize for this error and state that this does not change the scientific conclusions of the article in any way. The original article has been updated.

